# Acute toxicity analysis of Disarib, an inhibitor of BCL2

**DOI:** 10.1038/s41598-020-72058-8

**Published:** 2020-09-16

**Authors:** Shivangi Sharma, Kontham Kulangara Varsha, Susmita Kumari, Vidya Gopalakrishnan, Anjana Elizabeth Jose, Bibha Choudhary, Kempegowda Mantelingu, Sathees C. Raghavan

**Affiliations:** 1 Department of Biochemistry, Indian Institute of Science, Bangalore, 560012 India; 2 Department of Zoology, St. Joseph’s College, Irinjalakkuda, Kerala 680121 India; 3 Institute of Bioinformatics and Applied Biotechnology, Electronics City, Bangalore, 560100 India; 4 Department of Studies in Chemistry, Manasagangotri, University of Mysore, Mysuru, 570006 India

**Keywords:** Cancer, Drug discovery, Diseases

## Abstract

Small molecule inhibitors targeting BCL2 are explored as anticancer therapeutics. Previously, we have reported identification and characterization of a novel BCL2 inhibitor, Disarib. Disarib induced cancer cell death in a BCL2 dependent manner in different cancer cell lines and mouse tumor models when it was administered intraperitoneally. In the present study, using two syngeneic mouse models, breast adenocarcinoma (EAC) and Dalton’s lymphoma (DLA), we show that oral administration of Disarib resulted in significant tumor regression in a concentration dependent manner. Importantly, tumor developed in both female and male mice were equally sensitive to Disarib. Further, we have investigated the toxicity of Disarib in normal cells. Single dose toxicity analysis of Disarib in male and female mice after oral administration revealed no significant variations compared to control group for parameters such as body weight, food and water consumption and behavioural changes which were analysed for the entire period of study. Haematological and histopathological analyses also did not show any significant difference from the control groups. Thus, our results reveal safe use of Disarib as a small molecule inhibitor and provide the foundation for investigation of other preclinical studies.

## Introduction

BCL2 family of proteins play central roles in cell death regulation through the tight regulation of intrinsic pathway of apoptosis^1–3^. BH3 mimetics, the class of compounds that activate apoptosis by selectively binding and inhibiting anti-apoptotic BCL2 family proteins are explored extensively in the area of targeted anticancer therapies^4–6^. BCL2 is an antiapoptotic protein, which belongs to the BCL2 family that promotes cell survival by inhibiting the mitochondrial membrane pore formation^7–9^. Higher expression of BCL2 has been reported in several cancers including leukemia and lymphoma^8,10–12^. Identification of the important role of BCL2 in cancer development and chemo resistance, rendered it as an ideal target for cancer therapeutics^1,7^. Development of the BCL2 inhibitor, ABT199 has shown that, targeting BCL2 specifically can be a precise choice to avoid dose limiting toxicity^13,14^. Importantly, several BCL2 inhibitors are currently under clinical trials^15^ (https://clinicaltrials.gov/ct2/results?cond=BCL2+inhibitors&term=&cntry=&state=&city=&dist =). Results of ongoing trials indicate that inhibitors of BCL2 alone or in combination with other drug/s can be an important tool in cancer therapy^16–19^. Currently pan BCL2 inhibitors such asAPG1252 (NCT03387332, NCT04210037), BM1197, Obatoclax (NCT00684918, NCT00600964), TW-37, Gossypol analogues (NCT00848016, NCT00540722), Oblimersen (NCT00543075, NCT00062244), and selective BCL2 inhibitor such as S55746 (NCT02920697) either alone or in combination are being evaluated in clinical trials^15^ (https://clinicaltrials.gov/ct2/results?cond=BCL2+inhibitors&term=&cntry=&state=&city=&dist =). Apart from this ABT199 and ABT 263, ABT737 in combination are being examined under clinical trials for various cancers^15,20^.

We have previously reported the identification and characterization of a novel BCL2 inhibitor Disarib (Fig. S1), which caused effective tumor regression in multiple mice cancer models when administered through intraperitoneal (IP) route^15^. Disarib showed high specificity to BCL2 gene, but not to BCL-xL, BCL2A1 or other antiapoptotic genes of the same family and induced intrinsic pathway of apoptosis by disrupting the interaction of BCL2 and BAK^15,16^. Comparison of Disarib with ABT199, a FDA approved drug that is currently being used in clinic showed that Disarib has improved efficacy than ABT199, when tested ex vivo and in vivo^16,17^.

In the current study, we have explored tumor regression induced by Disarib, when treated through oral route, since this is the preferred route of administration to conduct clinical trial. Subsequently, we had taken a step toward the preclinical toxicological studies of Disarib as it holds the potential to be developed as an anticancer drug. Toxicity analyses of Disarib in Swiss albino mouse model revealed that higher doses of Disarib did not cause significant toxicity in the tested rodent model. Complete blood count (CBC) and histopathological analysis of kidney, liver and intestine were in line with the control mice. Toxicological data obtained from this study will help to select a dose for human therapies with minimal side effect.

## Results

### Oral administration of Disarib induces tumor regression in EAC and DLA mouse models

We explored the tumor regression property of Disarib by oral administration in two different syngeneic mouse tumor models. Tumor was induced in Swiss Albino mice either by injecting EAC cells or using cells derived from DLA tumor. These mice were treated with Disarib through oral route, once after visible tumor was observed in mice and the rate of tumor growth was recorded for a period of 22 days.

**Figure 1:**
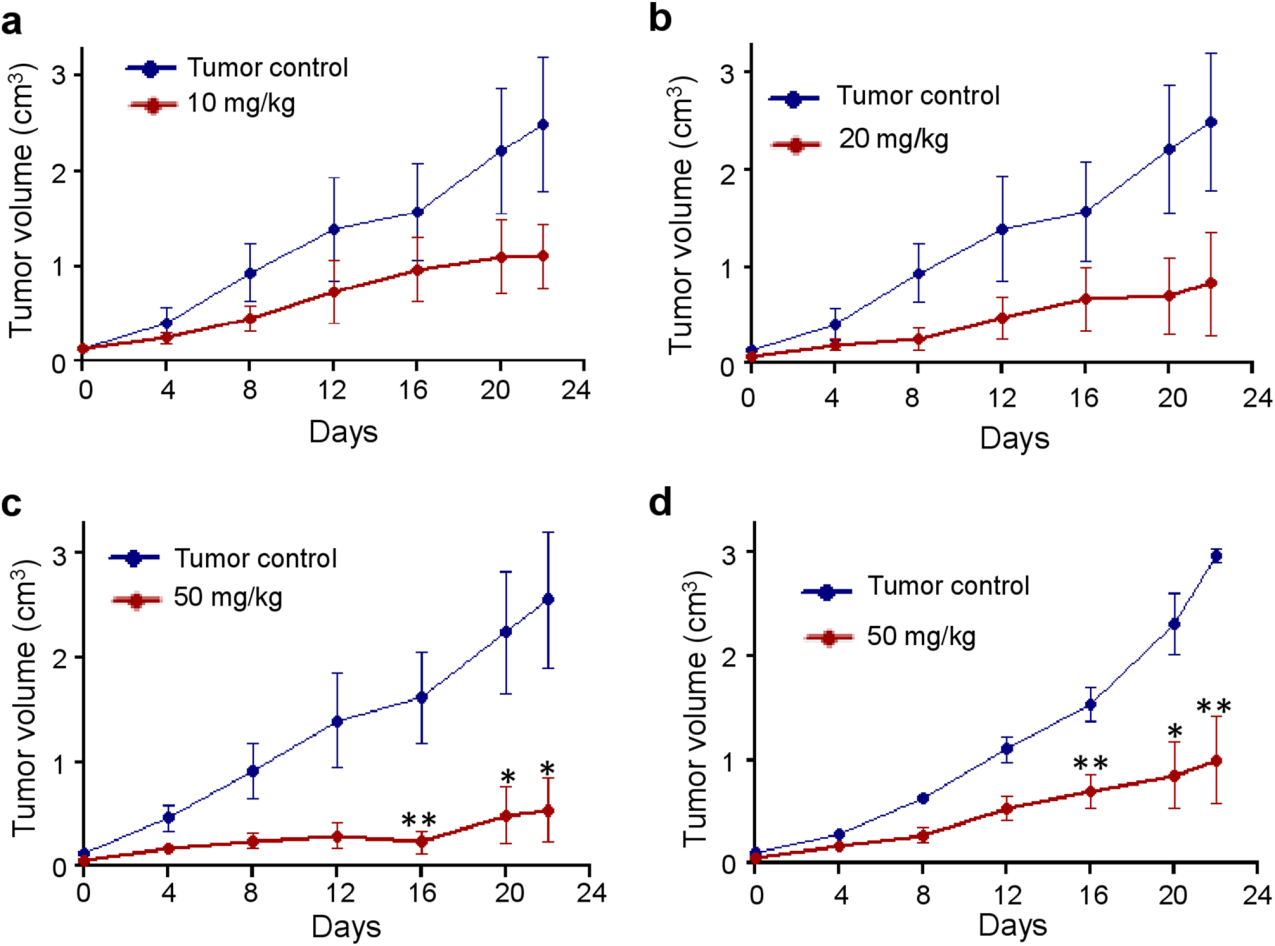
Effect of oral dose of Disarib on EAC tumor progression. Swiss albino mice bearing EAC were treated with multiple doses of Disarib as follows. (**a**) 10 mg/kg, b.wt., 6 alternate doses. (**b**) 20 mg/kg b.wt., 6 alternate doses. (**c**) 50 mg/kg b.wt, 14 continuous doses in female mice. (**d**) 50 mg/kg b.wt, 14 continuous doses in male mice (ns: not significant, *p < 0.05: **p < 0.005).

For the study, synthesis and characterization of Disarib was performed as described before^15^. In the first study, mice bearing EAC tumor was treated with 10 mg/kg or 20 mg/kg of Disarib, orally (6 doses, every alternate day). Results showed a significant reduction in tumor growth (Fig. 1a,b), which was comparable to the efficiency reported previously when Disarib was administered intraperitoneally^15,16^. Although reduction in tumor size was observed upon Disarib treatment, the inhibition in cancer cell proliferation was not complete as there was an overall tumor growth even after completion of treatment. To test whether Disarib treatment can result in complete regression of tumor growth we have orally administered Disarib (50 mg/kg) continuously for 14 days (Fig. 1c). Results showed complete regression in tumor size. These results were reproducible even when male mice were treated with continuous dose of Disarib (50 mg/kg) for 14 days, although the effect was pronounced in females (Fig. 1c,d). Thus, a decrease in the tumor volume was observed in all the treatment groups which demonstrate both the anti-tumor efficiency and the systemic absorption of Disarib in vivo. Interestingly, treatment with the 50 mg/kg body weight Disarib resulted in remarkable tumor size reduction and was approximately 4 times smaller than the control group.

**Figure 2:**
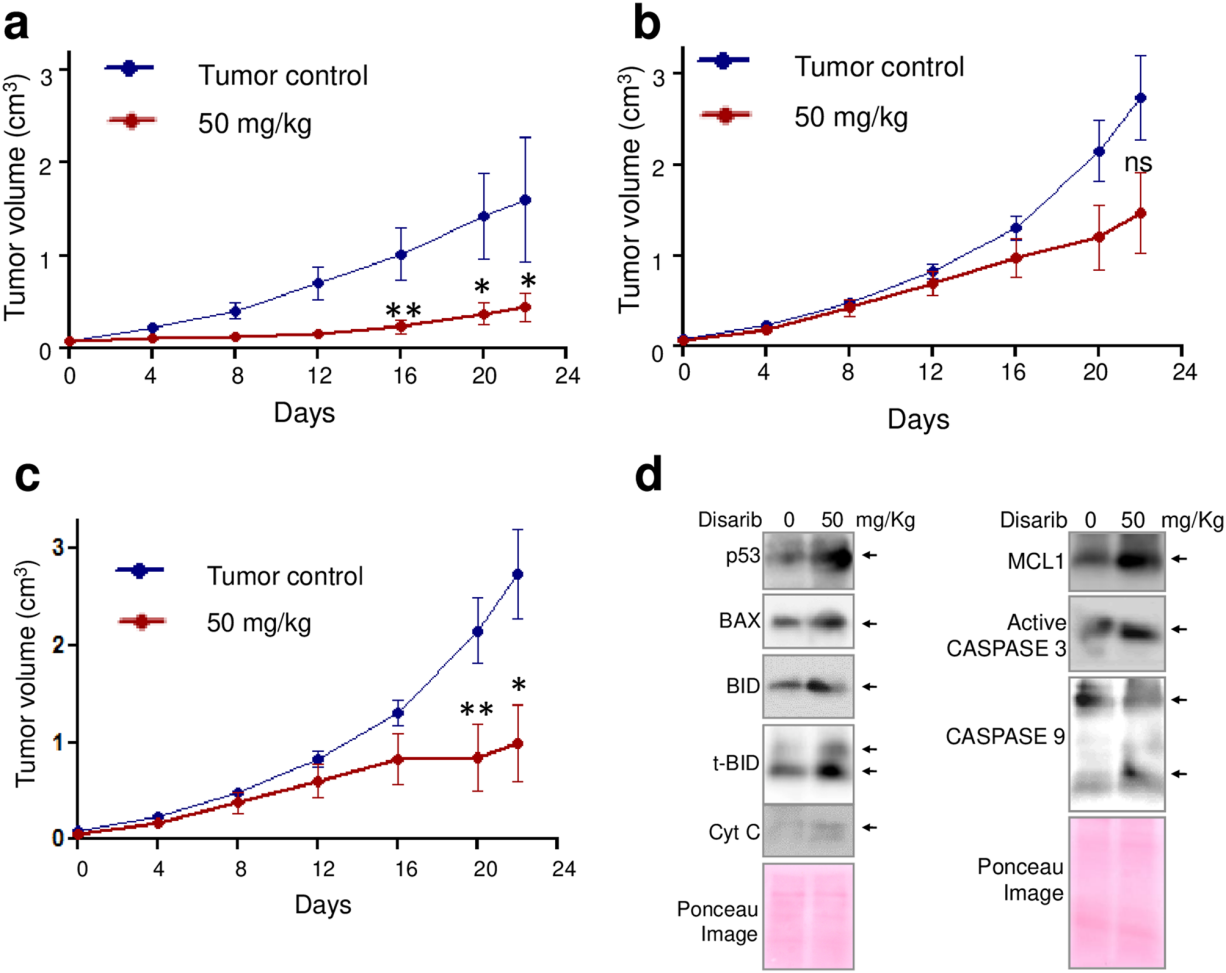
Effect of oral administration of Disarib on DLA tumor progression. DLA tumor was generated in male and female Swiss albino mice and were treated with multiple doses of Disarib on continuous days as follows. (**a**) 50 mg/kg b.wt., 14 continuous doses in female mice. (**b**, **c**) 50 mg/kg b.wt., 14 continuous doses in male mice including (**b**) and excluding (**c**) outliers. (**d**) Western blot showing evaluation of apoptotic markers in protein lysate prepared from DLA tumour cells from control (0) and Disarib treated (50 mg/kg) mice. The blots are derived from multiple gels and membranes were cut based on molecular weight. Refer also Fig. S2.

Efficacy of oral administration of Disarib was also tested in DLA tumour models generated in female (Fig. 2a) and male (Fig. 2b,c) mice (50 mg/kg, for 14 continuous doses). Among the male mice, three were not responding to the treatment compared to the remaining nine mice. The graphs depicting values from all the animals (Fig. 2b) as well as after removal outliers are presented (Fig. 2c). Results showed significant reduction in tumor growth upon oral administration in both the groups after 20 days of treatment (Fig. 2).

Thus, our results demonstrated that both syngeneic mouse models were sensitive to Disarib, when it was administered orally. It is also important to point out that the while the maximal dose used in the present study was 50 mg/kg, the dose used was up to 100 mg/kg when ABT199, the BCL2 inhibitor used in clinics, was tested in mice^18^.

### Disarib induces apoptosis in mouse tumor cells

Previously, extensive studies have shown that treatment with Disarib resulted in cytotoxicity in cancer cell line and in mouse tumor by inducing intrinsic pathway of apoptosis^16,17^. To confirm Disarib indeed induces apoptosis in tumour cells in vivo, when orally administered, immunoblotting was performed using lysate prepared from DLA tumor cells following treatment with Disarib (50 mg/kg) (Fig. 2d). Results showed increased expression of pro-apoptotic protein BAX, which are known to be the initiators of intrinsic pathway of apoptosis. Besides, upregulation of BID, cleaved BID and MCL 1 were observed upon treatment. Further, increase in the level of p53 and release of Cytochrome C was observed in the treated cells, substantiating the induction of apoptosis (Fig. 2d). Finally, activation of CASPASE 9 and CASPASE 3 was also observed (Fig. 2d). Thus, consistent to previous studies, we have observed activation of apoptotic markers in tumor cells, when Disarib was treated orally.

### Disarib treatment does not cause body weight changes in mice

Single dose toxicity experiments were performed according to guidelines of CDSCO, India^19–21^. IP and oral administration of Disarib was performed for toxicological evaluation with minimum five Swiss Albino mice per group. All the animals were observed for any change in physiology, behaviour and, food and water consumption. Results showed no distinguishable variation compared to control mice in different treated groups.

**Figure 3:**
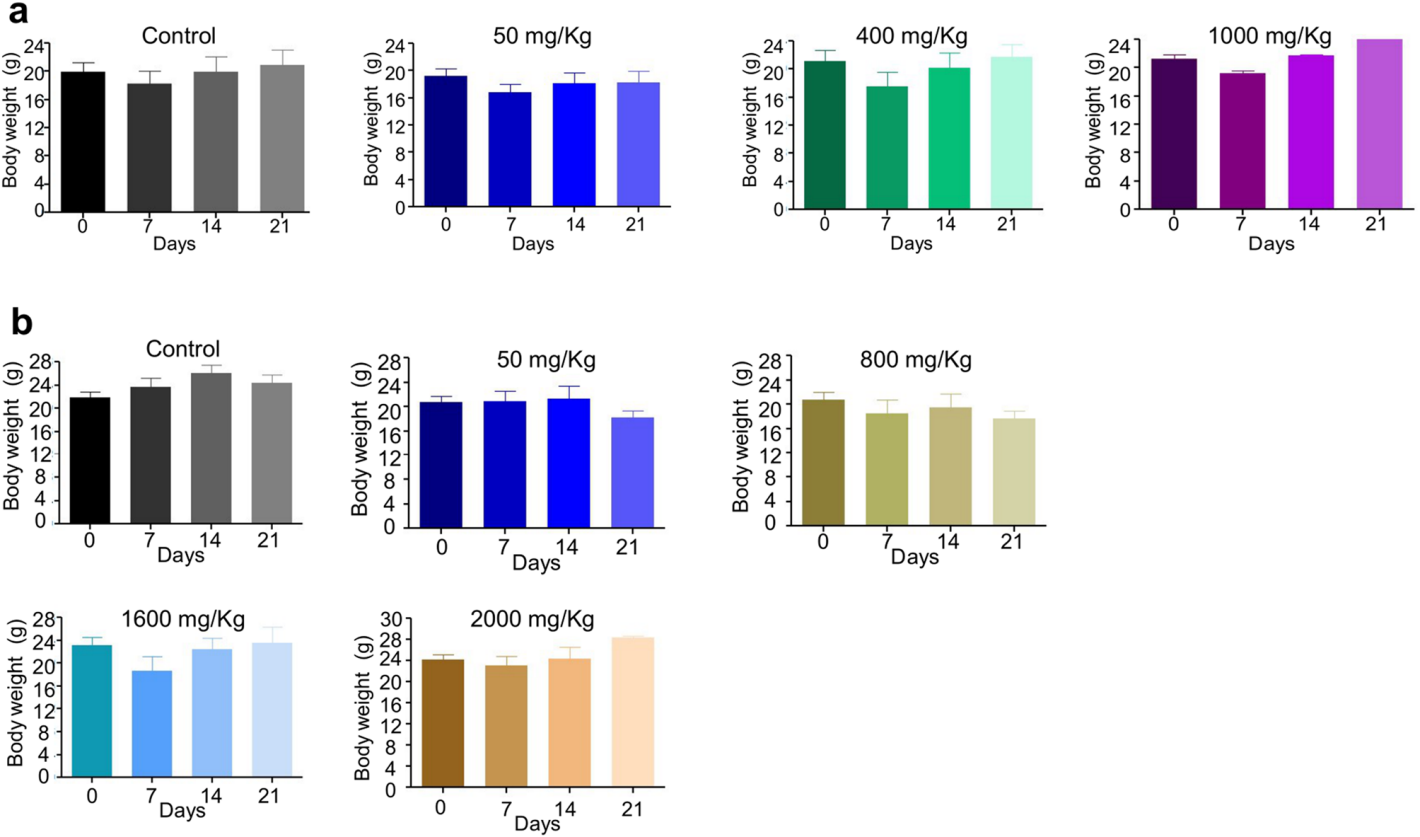
Systemic single dose toxicity studies of Disarib. (**a**) Body weight analyses of mice on 0, 7, 14 and 21 days after intraperitoneal administration of low, medium and high concentrations of Disarib (50, 400, 1,000 mg/kg b.wt. respectively). Mice treated with methyl cellulose served as vehicle control. (**b**) Body weight analyses of mice on 0, 7, 14 and 21 days after oral administration of Disarib (50, 800, 1,600 and 2000 mg/kg b.wt.).

Dose escalation study showed that a dose of 1,200 mg/kg body weight of Disarib was lethal for the mice when administered through intraperitoneal route (50, 400, 800, 1,000 and 1,200 mg/kg). However, administration of a dose of 1,000 mg/kg body weight of Disarib showed no significant toxicity and all the animals survived the study period (Fig. 3a). Further, dissection of mice treated with 1,200 mg/kg of Disarib revealed accumulation of unmetabolized chemical in the intraperitoneal cavity at this concentration (Data not shown). Based on this LD50 was determined to be ~ 1,100 mg/kg, when animals were treated through intraperitoneal route.

In contrast to this, administration of Disarib (50, 400, 800, 1,000, 1,200, 1,600 and 2000 mg/kg) through oral route did not show any significant change in body weight of mice even when the highest dose (2000 mg/kg) recommended by CDSCO (India) was used (Fig. 3b). Dissection of the animals showed no unmetabolized chemical even in the cases where highest dose was used (data not shown) and the LD50 was determined to be > 2000 mg/kg.

### Haematological parameters do not show any variation upon Disarib administration

**Figure 4:**
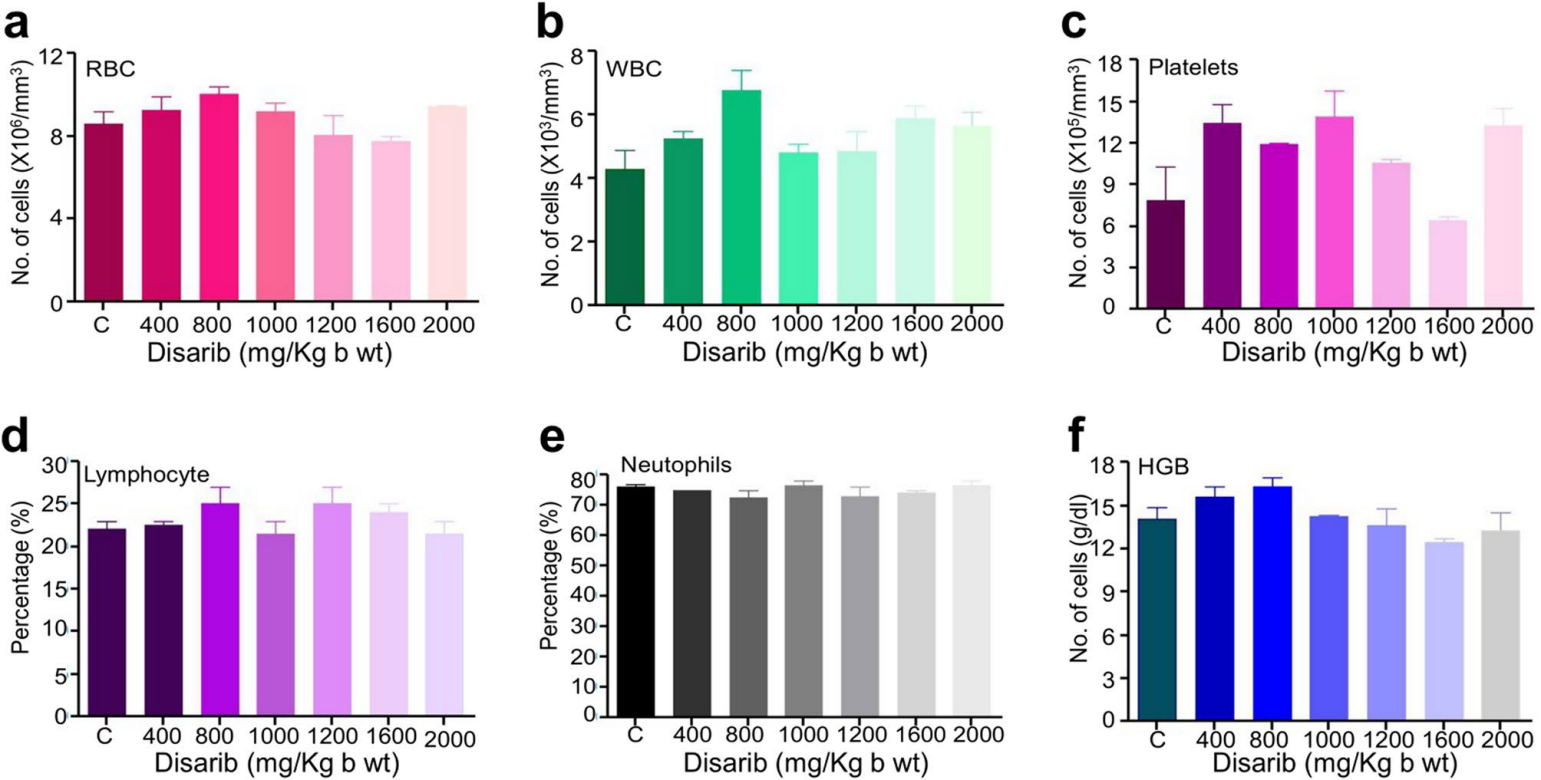
Haematological analyses following oral administration of Disarib in mice. Analysis of blood parameters among different doses of Disarib (400, 800, 1,000, 1,200, 1,600, 2000 mg/kg b.wt.) tested for toxicity. (**a**) RBC, (**b**) WBC, (**c**) Platelets, (**d**) Lymphocytes, (**e**) Neutrophils, (**f**) Haemoglobin.

We have evaluated complete blood count of mice from all orally fed groups after 14 days of Disarib treatment. The blood parameters analysed (RBC, WBC, platelets, lymphocytes, neutrophils and haemoglobin content) did not show significant variation compared to the control group and all the parameters were within range when compared with the control mice even for the highest (2000 mg/kg body weight) dose of Disarib (Fig. 4). Though there was an increase in platelet count in the treated groups, it was within the normal range^22,23^. Particularly, mouse treated with 1,600 mg/kg b.wt. showed platelets count lower than normal, however, the difference was not significant compared to control. The effect of IP administration of Disarib on haematological parameters was described before^16^ and showed no significant changes.

### Histological analysis does not show significant toxicity

**Figure 5:**
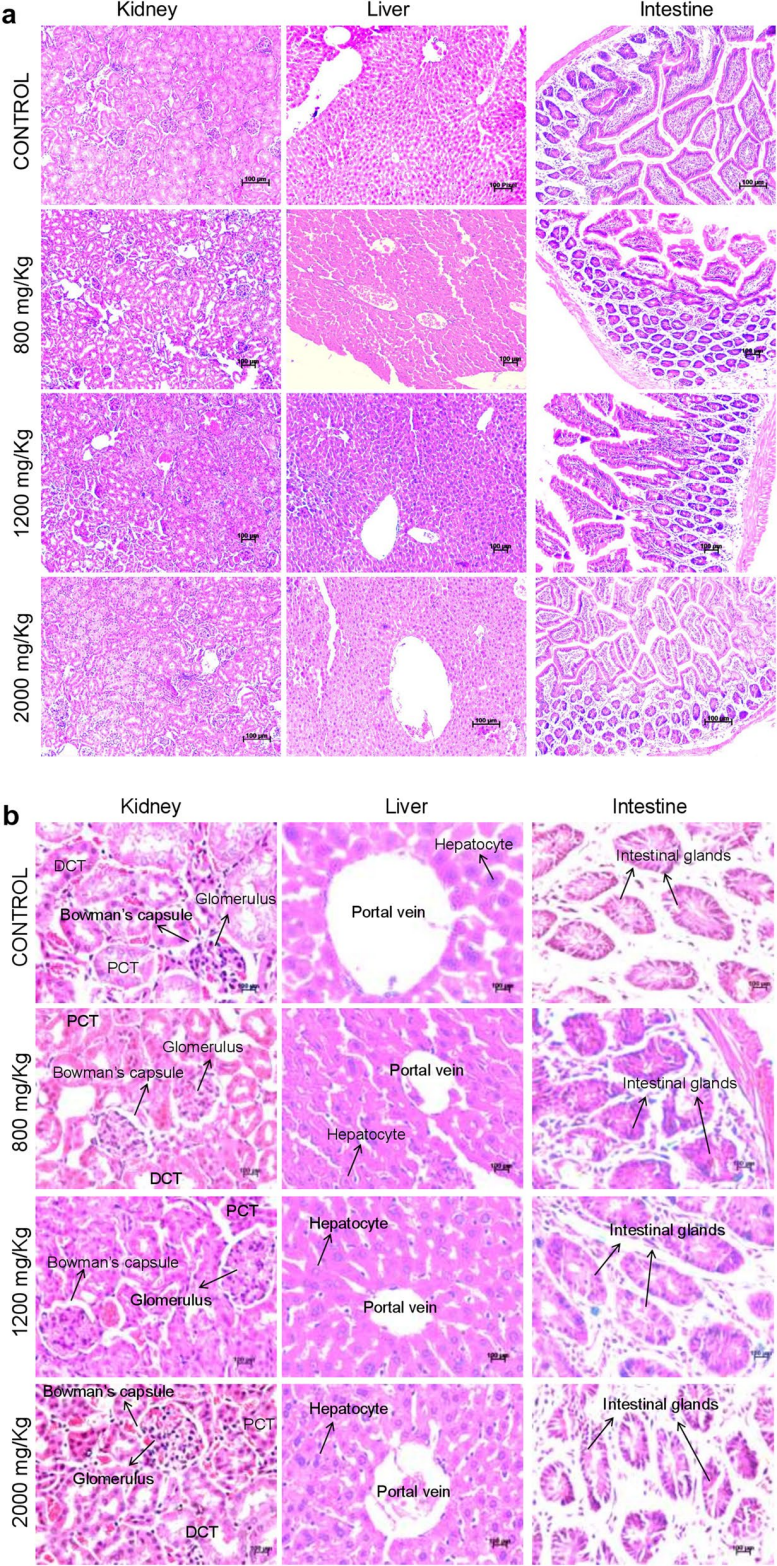
Histopathological analysis of different tissues of mice following oral administration of Disarib. (**a**, **b**) HE staining of tissues from control and Disarib administered mice (oral, 800, 1,200 and 2000 mg/kg b.wt.). Kidney, Liver, Intestine. Images shown are either with a magnification of 10X (**a**) or 40X (**b**). ‘DCT’ is Distal convoluted tubule, ‘PCT’ is Proximal convoluted tubule.

HE staining of the tissue sections derived from Disarib treated mice (800, 1,200 and 2000 mg/kg) showed no irregularity between the treatment and control groups (Fig. 5). The liver sections showed normal architecture and cellular integrity following Disarib treatment (Fig. 5). There was no hepatocellular cytoplasmic and nuclear alteration, which ascertained that there was no cellular level damage to the liver upon Disarib treatment (Fig. 5). The texture of glomeruli and renal tubules were normal and similar for the kidney sections in both treated and control animals (Fig. 5). Intestine section analysis also revealed normal pattern in both control and treated groups (Fig. 5). Thus, histopathology analysis of multiple organs revealed that Disarib treatment did not affect the normal architecture of the organs suggesting no toxicity upon treatment.

## Discussion

Previously we have described Disarib, as a small molecule inhibitor that can kill cancer cells in a BCL2 dependent manner both ex vivo and in mouse models when it was administered through intraperitoneal route. Importantly, head to head comparison of Disarib with ABT199, the clinically used BCL2 inhibitor showed better cytotoxicity and tumor regression efficacy when Disarib was used^16^ . Considering the target specificity and significant tumor regression potential of Disarib, in the present study we have evaluated non-clinical acute toxicity analysis in mouse. Since oral route of administration is favoured for a drug administration in humans, we have also explored the tumor regression efficacy of Disarib by oral route of administration.

In the present study we describe that Disarib can cause remarkable tumor regression when administered through oral route in two separate syngeneic mice models. Anti-tumor effect was significantly high with increasing Disarib concentrations and number of doses. While tumor cell proliferation was strictly regulated or inhibited by Disarib in the treated groups during the entire study period, it was highly proliferative in the case of control groups. Importantly, both male and female groups of animals responded to the treatment in more or less comparable manner. Further, there was no observable toxic effect in the treated mice even when the dose was increased to 50 mg/kg body weight and fed orally for 14 continuous days. Western blot analysis revealed upregulation of BCL2 family of proteins, particularly that are proapoptotic in nature. Thus, upregulation of multiple apoptotic BCL2 family proteins in the Disarib treated mouse tumor cells, in conjunction with activation of Caspases indicate the ability of Disarib to induce intrinsic pathway of apoptosis in vivo.

Single dose toxicity analyses with increasing concentrations of Disarib revealed no significant toxic effects. Although IP method of administration was lethal above 1,000 mg/kg body weight, a dose of 2000 mg/kg body weight did not cause death when orally administered. This difference may be explained as un-metabolized Disarib was found accumulated in the intraperitoneal cavity of the mice after dissection and suggests that higher dose of Disarib is not properly metabolized and absorbed into the body when given through IP route. More importantly, the oral treatment was nontoxic to the animals even when an amount of 2000 mg/kg body weight was administered. No change in appearance and behaviour, food and water consumption were observed for any of the treated groups from the control mice. Complete blood count analysis did not show significant variation in any of blood parameters investigated between control and treatment groups.

BCL2 inhibitors often cause dose-limiting thrombocytopenia. For example ABT-263 (Navitoclax), an inhibitor of BCL2 has shown clinical efficacy in many haematological cancers, but it causes thrombocytopenia due to interaction with other BCL2 family proteins like BCL-X_L_ and BCL-W^24^. Therefore, it is important to analyse platelets count after administration of high dose of BCL2 inhibitors. Interestingly, there was no reduction in the platelet count in any of the treatment groups under study, which was similar to ABT199, but unlike majority of BCL2 inhibitors studied in the literature^1^.

We selected kidney, liver and intestine for histological studies since these organs are involved in the direct absorption and excretion of drugs. Systemic absorption of the drug happens in the intestine followed by metabolism in the liver and excretion by the kidney. Analyses of these organ functions are vital in the determination of efficacy and safety of therapeutic drugs^25^. Importantly, there were no detectable variations between the control and treated animals in the architecture of tissues from the respective organs. This emphasizes that Disarib was metabolized inside the body without adversely affecting the functioning of organs.

From the tumor regression studies, we confirmed absorption of Disarib to blood after oral administration and its efficiency underlines its potential use as a specific anticancer drug. Single dose toxicity analyses of Disarib in mice proved its safe use as a small molecule inhibitor and the toxicity need to be tested in other rodents and non-rodents. Therefore, the current study provides the foundation for further preclinical and clinical trials.

## Methods

### Chemicals and reagents

Chemicals used in this study were procured from Sigma Aldrich, USA and SRL, India. Antibodies were obtained from Santa Cruz Biotechnology (USA) Cell Signaling Technologies (USA), Abcam (UK) and BD Biosciences (USA).

### Animals

Swiss albino mice purchased from the Central Animal Facility, IISc, Bangalore were maintained according to the guidelines of the Animal Ethical Committee. The animals were accommodated in polypropylene cages and provided standard pellet diet (Agro Corporation Pvt. Ltd. India) and water ad libitum. The animals were kept under regulated temperature and humidity with 12 h light/dark cycle. The experimental design and methods followed institutional guidelines and were approved by the institutional review board, Institutional Animal Ethics Committee, Central Animal Facility, Indian Institute of Science. Ethical committee approval no: CAF/Ethics/551/2017.

### Breast adenocarcinoma tumor model

Ehrlich ascites breast carcinoma (EAC) cells (1 × 10^6^) were injected to the left thigh region of Swiss albino mice and tumor was induced as described before^26,27^. Treatment was started once the tumor size was measurable. Two separate sets of experiments were conducted as pilot study with 6 doses i.e., 10 mg/kg (control, n = 5; treatment, n = 8) and 20 mg/kg body weight (control, n = 5; treatment, n = 8) of Disarib administered orally on alternate days. The subsequent study was conducted with 14 continuous oral doses of 50 mg/kg body weight Disarib in female mice (control, n = 5; treatment, n = 8), and in male mice with control (n = 5) and treatment group (n = 10). Tumor size was measured using vernier caliper and volume was calculated as described^16,26,28,29^ using the formula: V = 0.5 × a × b^2^, where ‘a’ and ‘b’ indicate major and minor tumor diameters respectively.

### Dalton’s lymphoma tumor model

Dalton's Lymphoma Ascites (DLA) cells (1 × 10^6^) were injected to the left thigh region of Swiss albino mice and tumor was induced^16,27,30^. Treatment was started once the tumor size was measurable. Two separate sets of experiments were conducted, with 14 continuous oral doses of 50 mg/kg body weight of Disarib to both male and female mice. The control group contained five animals and the treatment group had 12 animals. Tumor volume was measured as explained in Sect. "Breast adenocarcinoma tumor model".

### Western blot analysis

Tumor cells were collected on 25th day of experiment from control and Disarib treated (50 mg/kg, 14 doses) mice with DLA tumor and tumor samples were stored at -80ºC, until use. The tumor cells were thawed, washed twice in 1X PBS and cell lysates were prepared in RIPA buffer^30^. The lysates were resolved on 10–12% SDS page, transferred to PVDF membrane (Millipore, USA) and probed with antibodies^31,32^. Antibodies against MCL 1, CASPASE 9, CASPASE 3, BID (Santa Cruz, US), BAX (BD Biosciences, US), cleaved BID (Santa Cruz, US), Cytochrome C (Abcam, UK) and p53 (Santa Cruz, US) were used for the studies. The blots were developed using chemiluminescent substrate (Immobilon western, Millipore) and images were captured using gel documentation system (LAS 3,000, FUJI, JAPAN).

### Toxicity analyses

All the studies were performed in Swiss albino mice with 5 animals per group. Control group animals were fed with carboxymethyl cellulose which was used as the vehicle control. Single dose toxicity studies were designed based on guidelines from Central Drug Standard Control Organization (Schedule-Y-CDSCO, Appendix III), India. We have chosen complete blood count as one of the primary toxicity analysis parameter, since BCL2 inhibitors are reported to decrease the platelet count in blood^16^.

Disarib was given initially to single mouse and continued for the entire experiment based on the survival of treated animal for 24 h. For IP route of administration, firstly 50, 400, 800, 1,000 and 1,200 mg/kg body weight Disarib was administered in one mouse each and was observed for Disarib induced mortality for 24 h. In the treatment groups where no mortality was observed, full experiment was performed by injecting appropriate dose of Disarib in rest of the mice (n = 5). Since the mouse treated with 1,200 mg/kg body weight of Disarib did not survive after 24 h, dose up to 1,000 mg/kg body weight was selected for further study.

For the oral route of administration, dose upto 2000 mg/kg body weight was selected as it was the highest amount permitted by CDSCO, India for mouse toxicology studies. Apart from the concentrations used for IP mode, we increased the doses to 1,400, 1,600 and 2000 mg/kg body weight for oral dosing. Since all the mice survived after dose testing study using single mouse from each group when orally treated, complete experiment was performed as above using 5 mice per group (n = 5). Body weight of the animals was documented alternate days. Mice were then monitored for change in appearance, food and water intake along with any alteration in their activity compared to the control group for 24 h onwards. Survival of the animals was recorded for 21 days for IP and oral groups as per the guidelines and LD_50_ was determined.

### Haematology

Haematological analyses were performed for two animals from every group of orally fed animals after 14 days of observation. Blood was collected by heart puncture and complete blood count was performed (BC-2800-Mindray) by a veterinary pathologist. Blood parameters including RBC, WBC, platelets, HGB, lymphocytes and neutrophils were tested and compared with control mice values.

### Histopathology

After 14 days of Disarib oral administration, two mice from each group were dissected as detailed above and the internal organs were collected. The tissues were fixed in 4% paraformaldehyde and processed according to published protocols^27,33^. For the current study, we have chosen kidney, liver and intestine from the control and higher dose (800, 1,200 and 2000 mg/kg body weight) treated groups for histological analyses. A portion of each tissue was embedded in paraffin wax blocks and sectioned to 5 µm slices using rotary microtome (Leica Biosystems, Buffalo Grove, IL, USA). The paraffin was removed from the sections and stained with standard HE protocols as reported before^15,34–36^. The sections were mounted in DPX and imaging was performed by bright field microscope (Carl Ziess Axiovision, Oberkochen, Germany).

### Statistical analysis

All values in the figures are expressed as mean ± SEM. Statistical analyses were conducted by student’s t test using GraphPad Prism 5. Values were considered significant when P < 0.05.

## Supplementary information

Supplementary Information 1.
