# Supplementary material for: Acute toxicity analysis of Disarib, an inhibitor of BCL2
Source: Sci Rep. 2020 Sep 16;10:15188. doi: 10.1038/s41598-020-72058-8 (PMC7494999; doi:10.1038/s41598-020-72058-8)
Supplement: Supplementary file 1 — Supplementary Information 1. [file 41598_2020_72058_MOESM1_ESM.pdf]

Acute toxicity Analysis of Disarib, an Inhibitor of BCL2

Shivangi Sharma[1+], KonthamKulangara Varsha[1+], Susmita Kumari[1],

Vidya Gopalakrishnan[1,2], Anjana Elizabeth Jose[1], Bibha Choudhary[3]

KempegowdaMantelingu[4] and Sathees C. Raghavan[1*]

Supplementary Figure S1. Chemical structure of Disarib.

Supplementary Figure S2. Originals of western blot shown in Figure 2d. Original of western blots done for apoptotic markers in protein lysate prepared from DLA tumour cells from control (0) and Disarib treated (50 mg/Kg) mice. The blots are derived from multiple gels and membranes were cut based on molecular weight.

Figure S1

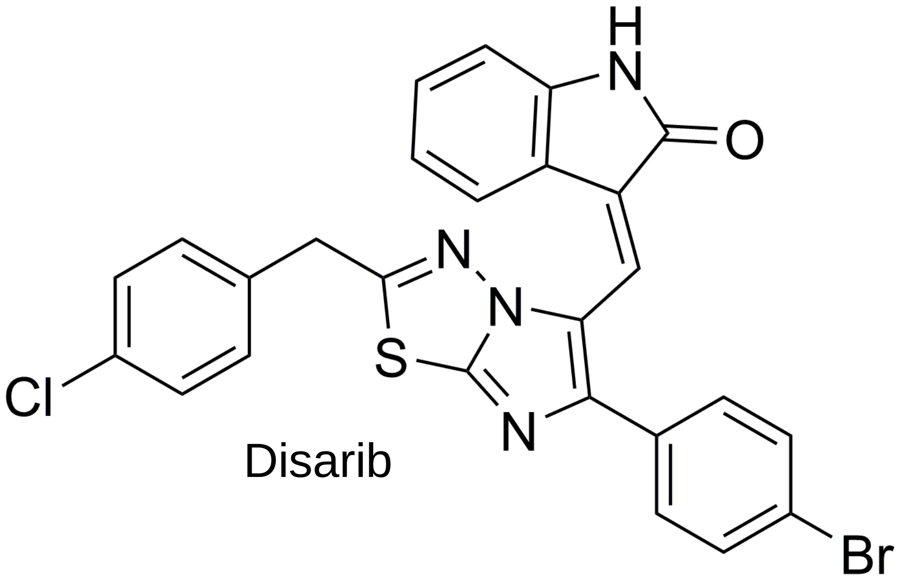

Disarib

Figure S2

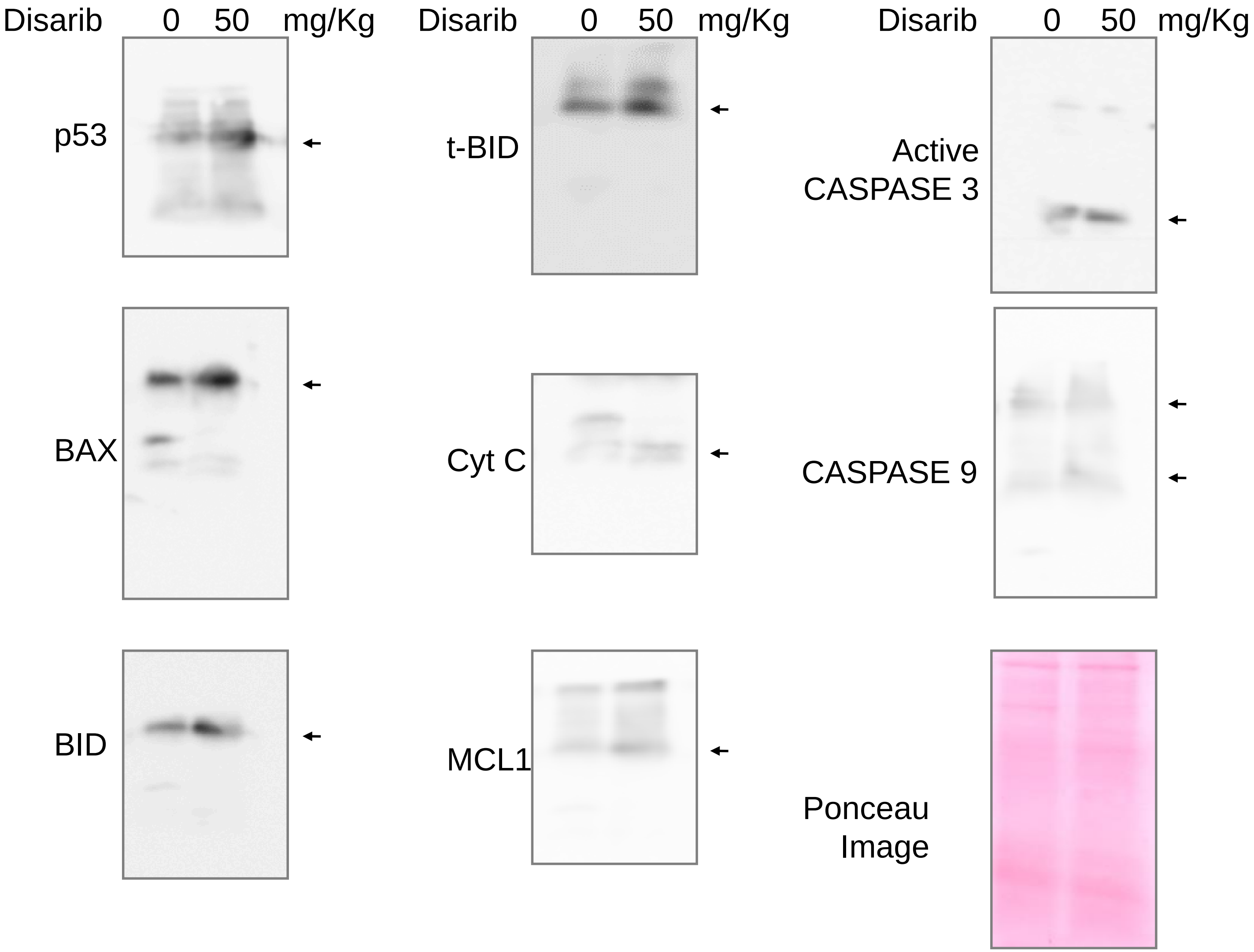
Disarib 0 50 mg/Kg
Disarib 0 50 mg/Kg
Disarib 0 50 mg/Kg
p53
t-BID
Active
CASPASE 3
BAX
Cyt C
CASPASE 9
BID
MCL1
Ponceau
Image
